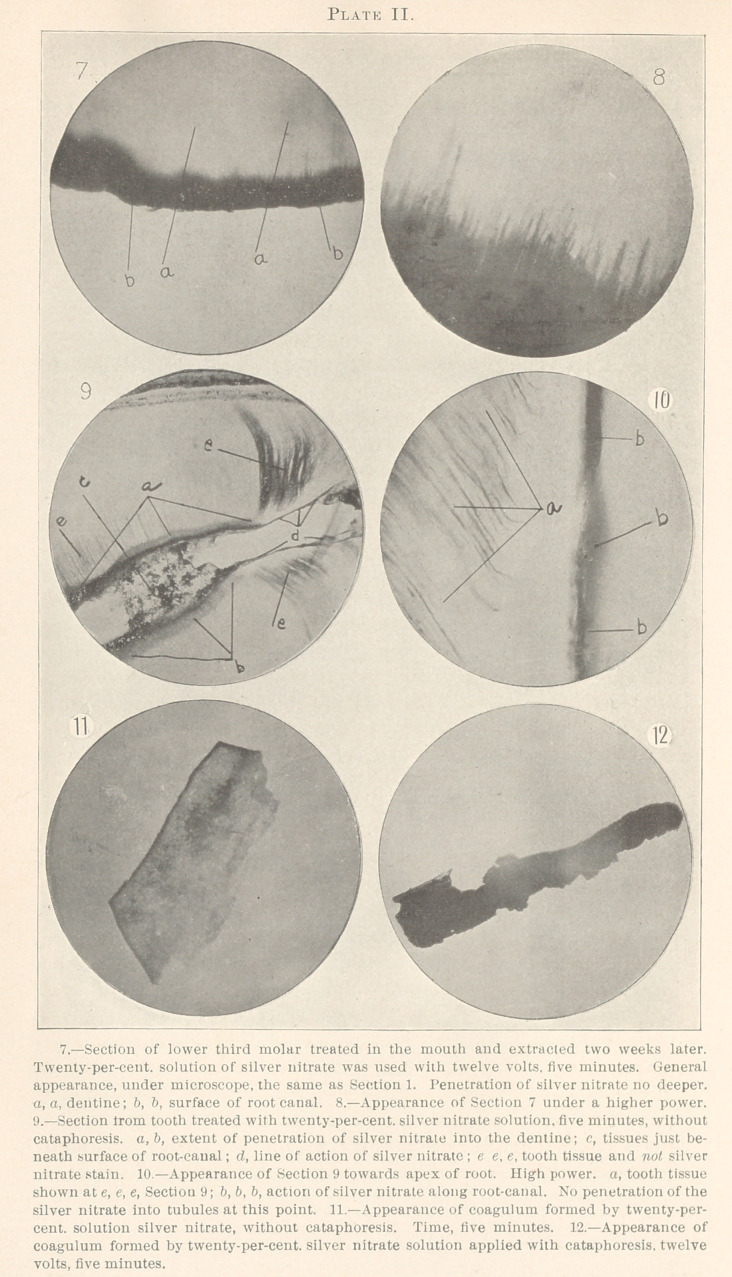# The Use of Silver Salts in the Treatment of Root-Canals

**Published:** 1897-08

**Authors:** L. P. Bethel

**Affiliations:** Kent, Ohio


### International Dental Journal.

VOL. XVIII. AUGUST, 1897. NO. 8.

### Original Communications.[1]

### THE USE OF SILVER SALTS IN THE TREATMENT OF ROOT-CANALS.[2]

BY L. P. BETHEL, D.D.S., M.D., KENT, OHIO.

THIS paper will serve as a sort of supplement to the one presented at the American Dental Association last August. In that I cited the results of experiments with silver nitrate as a lining for minute root-canals (not a filling, as some have taken it), showing that the silver nitrate, acting on the albuminous matter, formed an albuminate that sealed the mouths of the tubules, which would prevent ingress of putrescent fluids. (Specimens were exhibited at your November meeting.) I stated also that, "the object of the experiments was to find a means of treating root-canals that are too small to admit a broach, those branching or tortuous, those in flat-rooted teeth, etc., where the insertion of a protecting root-filling is a matter of doubt. If such canals are thoroughly lined with nitrate of silver, and it penetrates somewhat into the tubuli, as it does, there will probably be no subsequent trouble, though the root-filling should prove defective; and, indeed, it is a question whether root-filling would be necessary at all in the smaller canals." (Many of these we are unable to fill anyway.) In the preceding paper I mentioned also that those experiments were only the beginning of

---

[1] The editor and publishers are not responsible for the views of authors of papers published in this department, nor for any claim to novelty, or otherwise, that may be made by them. No papers will be received for this department that have appeared in any other journal published in the country.

[2] Read before the New York Institute of Stomatology, April 6, 1897.

a series in the direction of root-canal treatment. Since that time I have made some further observations.

Will silver nitrate, after being deposited in a root-canal, act as a permanent antiseptic?

Silver oxide being an inert substance, it seems reasonable to suppose that it does not. By way of experiment, a number of freshly extracted teeth were treated with silver nitrate cataphorically, and placed in sterilized test-tubes, where they remained one week. Each crown cavity was then filled with alkaline bouillon media and inoculated with a mixture of bacteria taken from a suppurating pulp and cultivated on glucose agar. These cultures were allowed two days to develop. Then from this inoculated media in each cavity I made a series of cultures on gelatin agar, glycerin agar, and glucose agar. All of the cultures, except the gelatin, were placed in the thermostat and kept at a temperature of about 37.5° C. (100° F.) for days, but no growth presented in any of the tubes, while the test-culture that I had made from the same tube of bacteria in bouillon had developed rapidly. This demonstrated that not only were bacteria within the tooth killed, but all those inoculated into the bouillon in the several cavities had been destroyed. After the bouillon media had been in the cavities one day it was found to be acid in reaction, although alkaline when inserted.

Why did the bacteria not develop? Nitrate of silver, uniting with the albuminous substance in the root-canal, forms a dense albuminate that must limit the action of the liberated nitric acid on the lime salts of the teeth. Then, during the application of the cataphoric current, the nitric acid will be attracted by the positive pole, being an anion, and therefore not driven into the tissues of the tooth like the metallic salt, which is a cation. So nitric acid will be found in the root-canal, and permeating the coagulum formed; then, when moisture is placed in that canal, the nitric acid passes into solution and we get an acid reaction. I think this explains why there was no growth of bacteria in the experiments just cited.

But Dr. Halsted, of Johns Hopkins Hospital, and Dr. Credé, of Berlin, Germany, have shown that when metallic silver is used as a dressing for wounds, "the products of the bacterial vitality oxidize the silver and enter into combination with the argentic oxide, forming argentic albuminates, which have strong antiseptic properties. It was found that the bacterial secretions entering into combination with the oxides are organic acids, pre-eminently lactic acid, and that the antiseptics which an infected wound, when dressed with the silver, generates of itself, is lactate of silver."

If these assertions are true, and practical experience seems to prove them so, it seems but reasonable to assume that, in case of subsequent infection of these root-canals treated with silver nitrate, as from an imperfect root-filling, etc., especially if the infecting bacteria be acid-producers, the acid excreted by the germs would enter into combination with the silver oxide lining the canal and form an antiseptic silver salt that would in turn destroy the bacteria that had gained entrance into the canal. That is, providing all conditions were favorable; but practical experience alone will demonstrate this.

For lining minute root-canals the seventy-five-per-cent. solution of silver nitrate has been used, as it is a more perfect conductor of electricity, and seems to penetrate the canals more readily than weaker solutions. For the larger canals, however, the twenty-per-cent. solution appears to answer all purposes. To determine the depth of penetration of silver nitrate into the dentinal tubules, a freshly extracted tooth was selected; remains of the putrescent pulp removed, the rubber dam was placed over the neck of the tooth, and the root inserted into absorbent cotton that had been placed in a rubber-plaster bowl and moistened with salt-water. The negative pole, or disk, had been laid face up in the bowl and the cotton packed over it so that there was perhaps an inch of moistened cotton between the cathode and the end of the root. Fifty-per-cent. sulphuric acid was pumped into the canal; the cataphoric current, twelve volts, was applied for a minute or two, using the negative electrode, or rather changing the wires at the binding-posts on the machine so that the platinum point would be negative and the disk positive; then the acid was neutralized with bicarbonate of soda and the canal rinsed with warm water and dried. Silver nitrate, twenty-per-cent. solution, was then pumped into the canal, the electric current applied (Wheeler's volt selecter) after reversing the wires so as to use the positive electrode, using twelve volts for five minutes, the electrode being of platinum wire and small enough to pass well into the root-canal. This detailed manipulation was carried out so as to conform as nearly as possible to the treatment of root-canals in the mouth, both for lining and for abscess. The tooth thus treated was allowed to dry out, and was then ground down and a microscopical specimen made. Various other specimens were made in this way and some from teeth treated in the mouth. (See Plates I. and II.) To get the full detail, these specimens should be seen through the microscope; but the microphotographs show the general appearance fairly well, except Sections 4 and 5, which should be as dark as the others. It will be observed that a dense coagulum clogs the mouths of the tubules (see Fig. 1), and as we follow the line of the tubules towards the circumference of the root, this coagulum grows less and less dense until only a few particles of silver oxide are seen scattered along in the tubules, and beyond this the normal tooth-structure remains. The extent of penetration is not more than one-sixth the extent of the tubules. In Section 4, under the microscope, the coagulum appears denser than in Section 1, and under a higher power (see Fig. 6), the granules of the deposited silver oxide appear larger than in specimens where twenty-per-cent. solution has been used.

Fig. 7 shows the appearance of a lower third molar that was treated in the mouth and extracted two weeks later. The general appearance under the microscope is about the same as Section 1, under both low and high power, when the same lenses are used. The illustrations do not represent the same magnifying power.

Section 9 shows the result of an application of twenty-per-cent. solution of silver nitrate for five minutes, but *without* using cataphoresis. In this section we observe the usual discoloration and some penetration into the tubules, but the coagulum along the canal-wall is not so dark or dense, and the silver oxide in the tubules not so impacted as in the specimens where cataphoresis was used. The middle portion of this section shows the tooth-structure just below the canal proper, while portions of the canal and side-walls are seen near either end. The difference in the appearance of Sections 11 and 12 is much more apparent under the microscope. Where cataphoresis was not used (see Fig. 11), the coagulum looks spongy, and is not so dark and dense as in Section 12, where cataphoresis was employed.

Some maintain that there is danger of forcing the silver nitrate through the whole extent of the tubules and causing injury to the peridental membrane. Perhaps this is possible if a very high voltage is used and the current applied for a long time; but in all of my experiments, and some have been with as high as forty volts, and in practical cases, I have not yet observed it. It is safe to affirm, I think, that this is impossible where from twelve to twenty volts only are used, and this seems to be about all the majority of patients can bear in root-treatment, and from four to five minutes time allowed.

In treating root-canals with silver nitrate, as much of the pulp as possible should be removed previous to the application, as the nitrate will then penetrate more readily. If small portions are left, however, they are thoroughly destroyed; at least this has been my observation in experiments outside the mouth. In experimenting, whole pulps have been thoroughly disintegrated.

Let us turn our attention to the practical phase of the subject. In treating root-canals, either lining canal or treating abscess, the following method is now employed: Adjust the rubber dam, remove all noxious matter possible from the canal; apply, with broach, either pyrozone or fifty-per-cent. sulphuric acid, or both, as circumstances indicate. When sulphuric acid is employed, the current is used one or two minutes with the negative electrode in the tooth, for acids are anions, and are driven from the negative towards the positive pole. The acid is then neutralized with bicarbonate of soda; then rinse the canal with tepid water. This precaution is taken, for silver nitrate forms a precipitate with sulphuric acid and bicarbonate of soda. The moisture in the canal is taken up with a cone of bibulous paper or cotton, twenty-per-cent. solution of silver nitrate is pumped into the canals with a broach, then with the positive electrode in the tooth, for all metallic salts are cations; apply the current, usually about twelve or fourteen volts, for from four to five minutes, according to conditions present. If desired to protect the crown cavity from discoloration, coat it with melted wax.

I am indebted to Dr. Henry Barnes, of Cleveland, for the history of the following cases that have occurred in his practice. By way of explanation I might add that these are but a few of the cases Dr. Barnes has treated with silver nitrate, and they represent not the most favorable but those cases that seemed most unfavorable before treatment.

Case I.—Miss H. E., aged twelve. Very putrescent case of abscess in the inferior left first molar. Could not even place cotton in the tooth without causing pain. Had treated with all known remedies but without avail, then in desperation used twenty-per-cent. silver nitrate solution cataphorically, twelve volts for five minutes. Filled roots immediately. Case got well at once and has been in perfect condition ever since. This treatment was August 15, 1896.

Case II.—R. W. D., aged twenty. Acute alveolar abscess in right superior second molar. Removed remains of pulp-tissue, washed out canals with electrozone, dried with cotton pellets and hot air, applied seventy-five-per-cent. solution silver nitrate cataphorically, twelve volts (Wheeler's selecter) for five minutes. Roots were filled at the same sitting with chloro-percha and gutta-percha. No soreness or after-trouble. Have treated three cases for this patient, all with acute abscesses caused by pulps dying under fillings. All were successful.

Case III.—Mrs. J. N., aged sixty. Inferior right first molar. Very putrescent condition of the affected tooth. Patient's health was not good and there was every indication of failure in treatment. Removed all putrescent material possible from root-canals, treated with electrozone, dried out, applied silver nitrate cataphorically, twenty per cent., for five minutes, and sealed the tooth. Soreness passed away and there was no after-trouble. Treated November 10, 1896.

Case IV.—Mrs. J. V. G., aged twenty-six. Case of putrescent pulp and blind abscess. Treatment the same as the others cited. In this case used seventy-five-per-cent. solution silver nitrate cataphorically. Filled root-canal at same sitting. Perfect success. No soreness or pain of any kind. Treated November 18, 1896.

Case V.—Mrs. N. S. W., aged fifty-eight. Putrescent pulp and blind abscess of superior right lateral incisor. The root was to be crowned. Treated with seventy-five-per-cent. solution silver nitrate cataphorically, and some was forced through the apex of root. Face in that location was swollen a little for two days but was not tender nor painful. Crown was applied four days later. No after-trouble. Roots and tissues have been in healthy condition ever since. Treated November 18, 1896.

In the following cases the nerve-tissue was not wholly removed from the teeth.

Case VI.—Patient, aged sixty. Inferior right molar tooth. Nerve had died under a large filling. Drilled into pulp-canal from buccal surface of tooth. Did not remove any of the nerve-tissue. Treated with oil of cassia for eight or ten days, but the tooth remained very sore and did not seem to improve under this treatment. Pus was still oozing out at the gum margins. In fact, it seemed as though the tooth was lying in a bed of pus. Nitrate of silver was used cataphorically and cavity sealed. Trouble ceased, soreness subsided, and tissues regained a normal condition. No further trouble.

Case VII.—Mrs. P., aged forty-five. Inferior right molar bearing a gold crown. Nerve died and tooth became very sore, but there was no swelling. Drilled through the morsal surface of the crown and was able to remove but a portion of the putrescent pulp-tissue. Treated several times with oil of cassia, but conditions did not improve. Applied twenty-per-cent. silver nitrate solution cataphorically. Trouble ceased, surrounding tissue became normal, and tooth has since given no trouble and is not at all tender on pressure.

Case VIII.—Patient, aged forty-five. This was very similar to the last case cited. A portion of the pulp near the apex of root could not be removed. Treated with silver nitrate, twenty-per-cent. solution, cataphorically, and there has been no subsequent trouble.

In treating more recent cases, Dr. Barnes has adopted the method outlined in this paper previous to citing the cases. He has lost but one case treated with the silver nitrate, and this he believes was due to having used sodium peroxide in the canal previous to the application of the nitrate, not knowing at that time that the two substances are incompatible and that the oxide of silver would be immediately precipitated on contact with the sodium peroxide, and before the current could be applied. All other cases treated have been cured by one application of the silver nitrate.

Regarding the incompatibility of silver nitrate with other much used antiseptics, I have observed that it forms a black flaky precipitate with sodium peroxide, a white granular precipitate with sulphuric acid, a white cloudy precipitate with bicarbonate of soda, and a white coagulate-looking precipitate with electrozone. No precipitate occurs with pyrozone or peroxide of hydrogen.

Before testing for incompatibility I treated some teeth experimentally, out of the mouth, with sulphuric acid and then immediately applied silver nitrate without neutralizing or rinsing out the acid. Upon filing the root and exposing the canal there appeared to be a perfect white root-filling extending from the apex about one-third the length of the root. When this was moistened with water, however, it immediately became a pasty mass. It was the precipitate caused by the union of the sulphuric acid and the silver nitrate, and prevented the penetration of the silver nitrate into that portion of the root-canal.

Thus incompatibility may lead to failure, and it is prudent to take the precaution of ridding the canal of these incompatible substances by neutralizing, rinsing, and drying before the silver nitrate is applied, if the best results are desired.

Dr. Credé, of Berlin, Germany, has recently brought out two new silver salts,[1] the lactate and citrate, which give promise of proving valuable additions to the list of dental antiseptics. They are white, odorless, tasteless powders, stable when kept from action

---

[1] Can be obtained from Schering & Glatz, 55 Maiden Lane, New York.

of light. The lactate is soluble in the proportion of one to fifteen parts of water, and the citrate in one to thirty-eight hundred parts of water. Dr. Credé states that they have no irritating or corrosive action on wounds, are non-poisonous, and do not destroy cellular tissue. These salts do not form a black coagulum with albuminous materials as will the nitrate of silver. In fact, they form a very slight coagulum, if any, but when left for some time in contact with albuminous material there appears a slight light-brown discoloration. Solutions of these salts are only feeble coagulators of egg albumen. They form no precipitate with sulphuric acid, pyrozone, or peroxide of hydrogen, but with sodium peroxide a black flaky precipitate is immediately thrown down. With electrozone a white cloudy precipitate appears, and with bicarbonate of soda solution a light cloudy precipitate presents.

To determine the action of the lactate solution on instruments, a bur was placed in a solution one to five hundred. The day following there appeared a grayish-brown precipitate on the instrument, but it was readily wiped off and the instrument did not appear to have suffered any. After this test I began a series of experiments to determine the efficiency of the lactate and citrate as instrument-sterilizers; but these were not carried out, for I found that when steel broaches, the blued portions of excavators, and other unpolished steel came in contact with the solution, a smoky, black precipitate began forming from the surface of the liquid, and the unpolished steel was soon blackened. I determined, however, that instruments infected in a bouillon culture of staphylococcus pyogenes aureus, allowed to air dry, next placed in the lactate solution, one to five hundred, for one minute, then planted on various media, showed a growth after two days. One and a half, two, and two and a half minutes' exposure all showed a growth, but the four minutes' exposure showed no growth on any of the media, thus demonstrating it to be an efficient germicide.

During the past four weeks the lactate, one to five hundred solution, has been employed in the treatment of a number of root-canals, but the time is yet too limited to give accurate data regarding its use in this connection. I may say, however, that it promises to be an efficient remedy. One case, treated three and a half weeks ago, was that of an abscessed lower molar. The nerve in the distal root was putrescent, but that portion in the anterior root was partially alive. When opened, pus oozed from the tooth. The tooth was loose and very sore. The canal was cleansed in the usual manner, one to five hundred solution of silver lactate applied cataphorically, twelve volts for five minutes; then a pellet of cotton saturated with the lactate solution was placed in the root as a dressing, and the tooth sealed. Soreness disappeared, and the tooth became firm. When opened a week later to see the condition of the root, the cotton was found to be perfectly dry, and the canal also, but the nerve-tissue in the anterior root was still alive. Had the application been silver nitrate, it would have effectually destroyed that remaining portion of pulp-tissue. In another case the lactate solution was forced through the apex of an abscessed root. The abscess was cured, but the patient experienced a slight burning sensation, which lasted for an hour or two and then subsided. This, according to Dr. Credé, is a characteristic of the salt, for he says, "It has no corrosive or irritating action upon wounds, but sometimes produces in sensitive ones a more or less strong burning sensation, varying in duration from several minutes to several hours."

In the case just cited there was a pocket in the gum on the lingual side of the tooth, extending almost to the apex of the root, and from which pus was exuding. A dressing of lactate solution was placed in this pocket, and the patient dismissed. No discomfort was experienced from this application, and the gum-tissue gradually tightened and resumed a healthy condition.

I have not made use of the lactate in pyorrhœa alveolaris, but believe it merits our attention in this connection.

In case it be required, in special abscess cases, to disinfect the contents of an abscess sac, or other conditions about the apex, the silver lactate solution can be forced into the apical space and the canal then treated with silver nitrate, if desired, for solutions of the two salts are compatible.

What is the actual condition of a root-canal in a pulpless tooth, and what do we have to overcome in treating that canal? I feel that I should not take time to go into detail regarding this condition, for this paper has already exceeded the limit. A few words, however, may not unduly tax your patience.

We do not find decay in a root-canal having a putrefying pulp, for the reaction of putrefaction is alkaline, and an acid reaction (fermentative process) is required for disintegration of the lime salts, which is a necessary antecedent to decay.

If there is no decay in a root-canal, do bacteria penetrate the tubules, and, if so, to what extent? I took from my stock of decalcified teeth a large number of those having abscessed roots. Sections, both longitudinal and cross sections, were cut from different portions of these roots, stained, and examined. In none of these was I able to find penetration of bacteria into the tubules, notwithstanding bacteria were present in the root-canal. Next, I selected a freshly extracted abscessed root. The crown had decayed almost entirely away, and the abscess on the apex of the root indicated a chronic case of long standing. By grinding this under water I was enabled to prepare a microscopical specimen of the entire length of the root-canal. (Unfortunately a good microphotograph of this section could not be obtained.)

Looking at this specimen with a low power, the surface of the canal appears irregular. With higher power the bacteria, stained violet, appear all along the length of the canal, in some places massed together, in others somewhat scattered, but conforming to the irregularities of the surface. No penetration of the bacteria into the tubules is observed until nearing the apex of the root, and here some have penetrated into the tubules to quite a depth.

It has for some time been a question in my mind whether the necessity for prolonged treatment, and, occasionally, failure to cure, were not due to failure in getting the antiseptic to the apex when applied as a dressing.

These, then, are the general conditions we meet in pulpless root-canals. What would be the result of an application of silver nitrate solution cataphorically to a root in this condition? It would combine with the albuminous bacteria, as well as the albumen in the tissues, to form in this location an insoluble albuminate, destroying the micro-organisms; it would penetrate into the tubules to a depth sufficient to destroy all noxious material, and then it would leave the mouths of the tubules thoroughly sealed. What more could we desire?

## Figures and Tables

**Plate I. f1:**
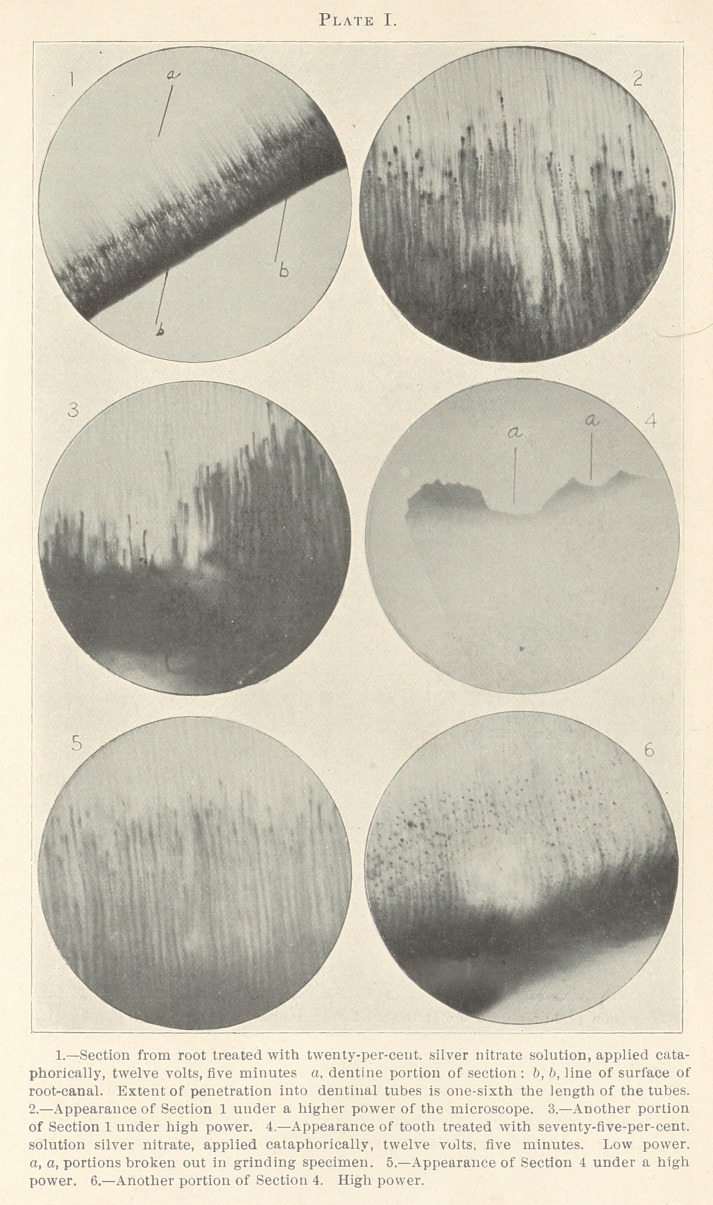


**Plate II. f2:**